# Low Serum Beta-2 Microglobulin Level: A Possible Biomarker for Sarcopenia in the Elderly Population

**DOI:** 10.3390/medicina60111879

**Published:** 2024-11-16

**Authors:** Ceren Kanat Sahin, Burak Mete, Hakan Demirhindi, Gülşah Yaşa Öztürk, Esra Ateş Bulut, Erkan Kozanoğlu, Gülçin Dağlıoğlu, Bülent Kaya, Ertuğrul Bayram

**Affiliations:** 1 Public Health Department, Faculty of Medicine, Çukurova University, 01250 Adana, Turkey; cerenkanat33@gmail.com (C.K.S.); demirhindi@cu.edu.tr (H.D.); 2 Department of Physical Medicine and Rehabilitation, Adana City Hospital, 1370 Adana, Turkey; gulsahyasaozturk@gmail.com; 3 Geriatrics Clinic, Adana City Hospital, Health Sciences University, 1370 Adana, Turkey; esraates@yahoo.com; 4 Department of Physical Medicine and Rehabilitation, Faculty of Medicine, Çukurova University, 01250 Adana, Turkey; ekozanoglu@yahoo.com; 5 Department of Medical Biochemistry, Faculty of Medicine, Çukurova University, 01250 Adana, Turkey; gulcinarikan@yahoo.com; 6 Department of Nephrology, Faculty of Medicine, Çukurova University, 01250 Adana, Turkey; bulentkaya32@gmail.com; 7 Department of Medical Oncology, Faculty of Medicine, Çukurova University, 01250 Adana, Turkey; ertugrulbayram84@gmail.com

**Keywords:** sarcopenia, beta 2-microglobulin, aged, early diagnosis

## Abstract

*Background and Objectives*: One of the most critical problems regarding sarcopenia is the difficulty of the diagnosis process. This study aimed to determine the prevalence and investigate the role of serum beta-2 microglobulin level as a biomarker for diagnosing sarcopenia. *Materials and Methods*: This nested case–control study was conducted between 2023 and 2024 on 251 older adults. Muscle strength was measured using a hand dynamometer, and muscle mass was assessed using the bioelectrical impedance method. Individuals with low muscle strength and low muscle mass were accepted as having definitive sarcopenia. *Results*: The mean age of the 251 older adults included in the study was 72.19 ± 6.11 years. The prevalence of sarcopenia in individuals aged 65 years and over was found to be 5.2%. Serum beta-2 microglobulin levels were statistically significantly lower in sarcopenic participants compared to the control group (*p* = 0.002). The optimal cut-off value for serum beta-2 microglobulin level was 2.26 mcg/mL, and values lower than this point were found to be diagnostic for sarcopenia. Regarding the cut-off value, the sensitivity was 92.31% and the specificity was 80.77%, the positive predictive value was 70.59%, the negative predictive value was 95.45%, the Youden index was 0.731, and the area under the curve value was 0.901. Individuals who had beta-2 microglobulin levels below 2.26 mcg/mL were found to have a 10.75 times higher risk of sarcopenia. *Conclusions*: A low serum beta-2 microglobulin level has the potential to be an important candidate biomarker for the diagnosis of sarcopenia.

## 1. Introduction

Sarcopenia is a skeletal muscle disease characterised by progressive loss of skeletal muscle mass and muscle function, which can lead to falls, weakness, functional decline, and ultimately death [1]. Muscle strength and mass fluctuates throughout human life. Muscle strength and mass peak between 30 and 40 years of age, and muscle loss increases linearly thereafter [2]. The prevalence of sarcopenia has varied over the years. One of the most important reasons for this is the difference in the measurement methods used and the change in diagnostic criteria over time. There is no gold standard method for diagnosing sarcopenia, and there are differences in the interpretation of methods and the evaluation of results [3]. This leads to difficulties in the diagnosis of sarcopenia. The use of a biomarker in the diagnosis of sarcopenia may contribute to a more objective evaluation and reduction in differences.

Beta-2 microglobulin is a low-molecular-weight protein with a molecular weight of 11,800 Da. It is found on the surface of nucleated human cells. It is part of the MHC (major histocompatibility complex) class I family. This protein is produced at a constant rate under physiological conditions. It accumulates in the circulation of patients with kidney diseases, immunodeficiency, and autoimmune or haematological diseases. Studies have reported it as a prognostic marker for some diseases such as non-Hodgkin’s lymphoma and HIV [4,5]. Beta-2 microglobulin has been determined by some researchers to increase in frail elderly people especially [6,7]. Both frailty and sarcopenia are the end pathways of many pathological processes. In frailty, skeletal muscle reduction is an important feature and includes low physical function as a component. We noticed the lack of literature on the role of beta-2 microglobulin in sarcopenia and decided to examine the role of beta-2 microglobulin in sarcopenia and its diagnosis. The aim of this study was to determine the prevalence of sarcopenia in a population aged 65 years and over and to examine the role of serum beta-2 microglobulin as a biomarker in diagnosing sarcopenia.

## 2. Materials and Methods

### 2.1. Research Type and Ethics

This nested case–control study was conducted between 2023 and 2024 in Çukurova University Faculty of Medicine’s Department of Public Health, including the affiliated Family Health Centres in the Application and Research Area of the Department, Çukurova University Balcalı Hospital’s Department of Physical Medicine and Rehabilitation, Adana City Hospital’s Department of Physical Medicine and Rehabilitation, and the Department of Geriatrics in Adana, Turkey.

### 2.2. Determination and Selection of Sample Size

The minimum sample size to be reached in this study was calculated as 249 people with a reference prevalence of 16% [8], assuming a type 1 error of 0.05, a power of 0.8, and an effect size of 0.07 in a double-tailed test. A total of 791 older adults were interviewed in Adana City Hospital’s Physical Medicine and Rehabilitation outpatient clinic and service, 113 in Çukurova University Faculty of Medicine’s Physical Medicine and Rehabilitation outpatient clinic, 127 in Adana City Hospital’s Geriatrics outpatient clinic, and 70 in Family Health Centres in the Application and Research Area affiliated with Çukurova University Faculty of Medicine’s Department of Public Health. The people in the sample were contacted within 12 months. In total, 1364 older adults were interviewed and 253 people were recruited based on the inclusion and exclusion criteria; among them, two people were excluded from the study due to errors in body composition measurements. The study was completed with 251 people. The convenience sampling method was used for the sampling. An informed consent form was signed, and only those who participated voluntarily were included in the study. The sampling was terminated when the determined sample size was reached.

**Inclusion criteria:**

Being over 65 years old;

Volunteering to participate in the study.

**Exclusion criteria for muscle mass measurement (Bioelectrical impedance analysis):**

Presence of a pacemaker;

Lymphoedema;

Joint prosthesis;

Prolonged immobilisation;

Hemiplegia;

Paraplegia;

Malnutrition.

**Determination of case and control group:**

In patients with a definite diagnosis of sarcopenia, a control group was selected by frequency and group matching.

**Case group (*n* = 13): “Definite sarcopenia”** [9]**.**

Definite sarcopenia = low muscle strength + low muscle mass.

**Low muscle strength:**

Handgrip strength test result <16 kg in females;

Handgrip strength test result <27 kg in males.

**Low muscle mass:**

Appendicular skeletal muscle mass <15 kg in females;

Appendicular skeletal muscle mass <20 kg in males.

**Control group (n = 26):**

It consisted of people over 65 years of age without sarcopenia.

**Matching:** Two methods were used for matching and determining the control group.

**Frequency matching (2:1):** Number matching was performed and two controls were selected for each case.

**Group matching:** The control group was matched with the cases according to sex, age, chronic disease status, and frailty status.

Factors that have a confounding effect on beta-2 microglobulin levels were taken into consideration in both case and control group selection. Serum beta-2 microglobulin levels were analysed in 100 individuals without the identified factors.

**Exclusion criteria for beta-2 microglobulin** [7]**:**

Identified malignancy;

Stage 3 or 4 congestive heart failure;

Stage 3 or 4 chronic kidney disease;

Chronic liver disease;

Obesity (BMI ≥ 35 kg/m^2^);

Autoimmune disease;

Use of anti-inflammatory agents, antibiotics, or immunosuppressive drugs;

Long-term fracture history/immobility;

Malabsorption/nutritional disorder.

### 2.3. Data Collection

Patients who agreed to participate in this study in outpatient clinics or health centres were informed about the study, signed an informed consent form, and completed a face-to-face questionnaire form. The questionnaire form consisted of a sociodemographic section, a primary care physical activity questionnaire, the frailty scale “FRAIL”, and questions from the sarcopenia screening test, “SARC-F”. The questionnaire was applied to the patients face-to-face by the researchers. The three-day nutritional information form was explained to the patients, who completed the questionnaire form and were asked to record their three-day nutritional information and bring the completed form to the next appointment, or send it via telephone (by WhatsApp). Muscle strength and muscle mass were measured in all patients who completed the questionnaire. Patients with low muscle strength and low muscle mass were considered to have definite sarcopenia, and a blood sample was taken from appropriate patients to check their beta-2 microglobulin levels.

### 2.4. General Practice Physical Activity Questionnaire (GPPAQ)

This questionnaire was first used in the United Kingdom in 2002 to assess the mobility level of adults in primary care [10]. Turkish validity and reliability tests were performed by Noğay and Özen (2019) [11]. It is a questionnaire consisting of seven questions under three main headings. The questionnaire is applied to adult individuals and classifies them as “mobile”, “moderately mobile”, “less mobile”, and “immobile”.

### 2.5. Sarcopenia Screening Test (SARC-F Scale)

The translation of the SARC-F scale into Turkish for individuals over 65 years of age was performed by Kış and Karaca in 2021 [12]. This questionnaire consists of five components: strength, assistance in walking, getting up from a chair, climbing stairs, and falls. These components were chosen to reflect the changes in health status associated with the consequences of sarcopenia. The SARC-F scale has a minimum score of 0 and a maximum score of 10. A score of 0–3 is considered healthy and a score of 4 and above is considered symptomatic.

### 2.6. Frailty Scale (FRAIL Scale)

The Turkish validity and reliability study of the FRAIL scale was performed by Hymabaccus et al. in 2023. The scale has five sub-dimensions: fatigue, endurance, ambulation, illness, and weight loss. Each sub-dimension reveals a score of 0 or 1. The total score varies between 0 and 5, with “0 = normal”, “1–2 = prefrail”, and “3–5 = fragile” [13].

### 2.7. Nutrition Information Form

A three-day nutritional information form was used to evaluate food intake. The three-day nutrient values were calculated with BeBiS 9 (Nutrition Information System) software and averages were taken. BeBiS is a computer software programme containing more than 130 nutrient analyses of over 20,000 foods.

### 2.8. Muscle Strength Measurement

Muscle strength was measured with a calibrated JAMAR brand hand dynamometer. Muscle strength was measured three times at 30-s intervals for both hands with a 90-degree elbow position and the highest value was recorded.

### 2.9. Body Composition Measurements (Bioelectrical Impedance Method-BIA)

The bioelectrical impedance method is based on the principle that different body components show different resistance to electric current. Lean tissues show low resistance and good conductivity to the passage of electric current, while fat, bone, and skin tissues show high resistance and low conductivity. After the height of the patients was measured, the Tanita BC 601 Practico Scale device (TANITA EUROPE B.V. Amsterdam, The Netherlands) was used for body composition measurements [14].

### 2.10. Measurement of Serum Beta-2 Microglobulin Level

Blood samples were collected from the participants in a Becton Dickinson vacuum-gel tube for the measurement of serum beta-2 microglobulin level. The blood samples were centrifuged in an NF1200R model device at 4000 rpm for 10 min on the same day. The separated serum was divided into Eppendorf tubes and stored at −80 °C. When the targeted number of samples were reached, all samples were transferred to the refrigerator at 4 °C, 24 h before being used. The samples were brought to room temperature immediately 30 min before the study. Serum beta-2 microglobulin level was measured manually by a sandwich enzyme-linked immunosorbent assay (ELISA) method with a BTLAB Mikroelisa test kit and measured according to the kit protocols.

### 2.11. Statistical Analysis

IBM SPSS Statistics 20 (SPSS, Inc., Chicago, IL, USA) and Jamovi (ver. 2.3.28) software were used for data analyses. The Shapiro Wilks test was used as the normal distribution test. Parametric tests (Student’s *t*-test, Pearson correlation test) were used for the analyses of data conforming to normal distribution, non-parametric tests (Mann–Whitney U test, Spearman correlation analysis) were used for analyses of data not conforming to normal distribution, and a chi-square test was used for the comparison of categorical data. Logistic regression analysis and multivariate linear regression analysis were used in multivariate analyses. ROC analysis was used to determine the optimum cut-off value for beta-2 microglobulin. Sensitivity (%), specificity (%), positive predictive value (PPV) (%), negative predictive value (NPV) (%), Youden index (sensitivity + specificity − 1), and area under the curve (AUC) metrics were used to determine the optimum cut-off value. *p* < 0.05 was considered statistically significant.

## 3. Results

The mean age of 251 older individuals included in our study was 72.19 ± 6.11 (min. 65; max. 91); among them 71.3% were in the 65–74 year age range. Of the participants, 62.2% were female and 82.1% had at least one chronic disease. The participants were tested for physical activity using the General Practice Physical Activity Questionnaire (GPPAQ), and it was found that the physical activity level of all participants was sedentary (Table 1).

**Figure 1:**
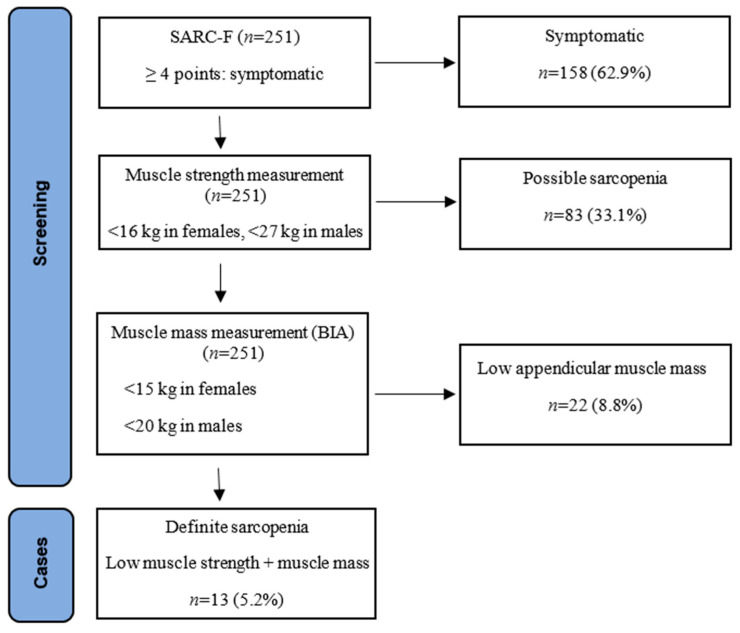
Sarcopenia screening process [9]. SARC-F: sarcopenia screening Test; BIA: bioelectrical impedance analysis.

As a result of the sarcopenia screening test of the 251 older adults included in this study, it was determined that 62.9% were symptomatic, 33.1% had low hand grip strength, and 8.8% had low muscle mass. The prevalence of sarcopenia was found to be 5.2% in our study (Figure 1).

In the matched case–control group, serum beta-2 microglobulin levels were found to be statistically significantly lower in elderly sarcopenic participants (Table 2).

**Figure 2:**
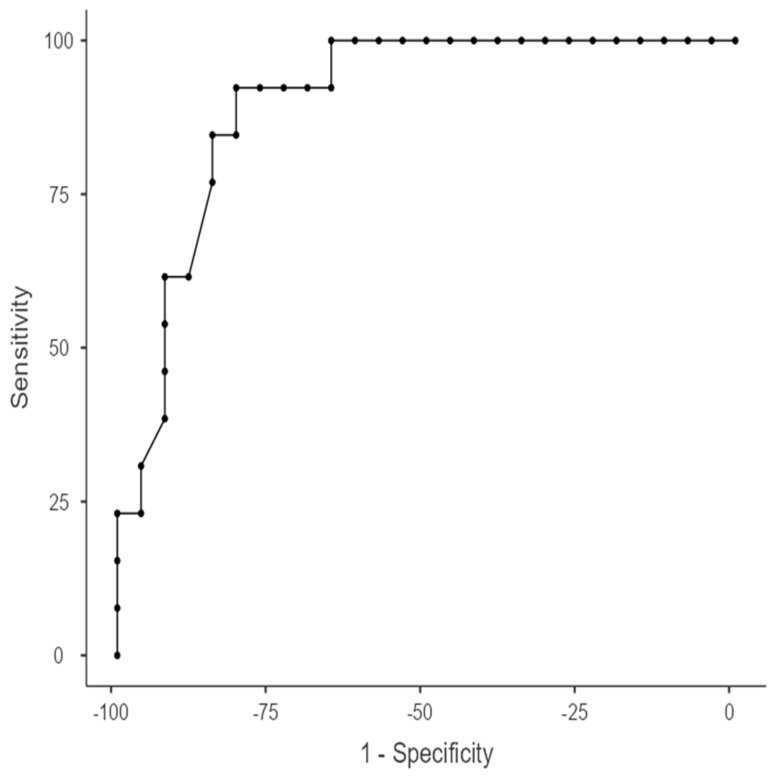
Area under curve for beta-2 microglobulin.

In the ROC curve analysis performed to evaluate the place of serum beta-2 microglobulin level in sarcopenia classification, it was found that the area under the curve was significant (AUC = 0.901, *p* < 0.001) (Figure 2). In our study, the optimum cut-off value for beta-2 microglobulin was determined as 2.26 mcg/mL. Beta-2 microglobulin values lower than 2.26 mcg/mL were found to be diagnostic (classificatory) for sarcopenia. For the optimal cut-off value, sensitivity was found to be 92.31%, specificity 80.77%, positive predictive value 70.59%, negative predictive value 95.45%, Youden Index 0.731, and area under the curve (AUC) 0.901 (Table 3, Figure 2).

Logistic regression analysis to predict the risk/probability of sarcopenia was found to be significant (Omnibus test, *p* = 0.003), the accuracy of the model was 87.4%, and the goodness of fit (Nagelkerke R Square = 0.244) was adequate. The independent variables of the model were age, sex, frailty score, average daily dietary energy, protein, fat and carbohydrate intake, chronic disease status, and beta-2 microglobulin level (risk category < 2.26 mcg/mL). Among the variables included in the model, each unit increase in age increased the risk of sarcopenia by 1.3 times, and serum beta-2 microglobulin levels less than 2.26 ug/mL increased the probability of sarcopenia by 10.75 times (Table 4).

## 4. Discussion

Sarcopenia is an increasing and often undiagnosed health problem. Its prevalence in the older population varies depending on sex, age, pathological conditions, and diagnostic criteria [15]. In our study, the prevalence of sarcopenia in older adults was found to be 5.2%, and serum beta-2 microglobulin level was found to be a powerful test for sarcopenia classification. The optimum cut-off value was calculated as 2.26 mcg/mL, and the values below this were found to be diagnostic for sarcopenia. For this cut-off value, sensitivity was 92.31%, specificity 80.77%, positive predictive value 70.59%, negative predictive value 95.45%, Youden index 0.731, and the area under the curve 0.901. The probability of sarcopenia was 10.75 times higher in individuals with beta-2 microglobulin levels below 2.26 mcg/mL.

Loss of muscle mass shows a linear increase after the 40s, and the loss of 8% per decade after the fourth decade increases to 15% after the age of 70, and the total loss of muscle mass can reach 50% in the 80s [16]. In a study by Batsis et al., in patients aged 60 years and older, the mean age of sarcopenic patients was found to be 70.5 years in males and 71.6 years in females [17]. In these studies, it was found that the prevalence of sarcopenia increased with age in both sexes, and the decrease in muscle mass was associated with age in both sexes [18,19]. In our study, it was found that the mean age of sarcopenic patients was higher, and the increase in age was associated with a decrease in muscle mass.

The prevalence of sarcopenia varies over time due to the change in the cut-off values used in the diagnosis of sarcopenia, and prevalences are reported to be between 5 and 50% [15]. In a meta-analysis by Shafiee et al. (2017), the prevalence of sarcopenia among males and females over 60 years of age was estimated as 10% [20]. In a systematic review by Papadopoulou et al. (2019), the prevalence of sarcopenia was reported to be 11% in men and 9% in women [21]. In a meta-analysis by Petermann et al. (2022), which included studies using different classifications and cut-off points, the prevalence of sarcopenia was reported to be between 8 and 36% in individuals under 60 years of age and between 10 and 27% in individuals aged 60 years and over. The prevalence of sarcopenia was reported to be higher in males when the European Working Group on Sarcopenia in Older People 2 (EWGSOP2) criteria were referenced (11% vs. 2%), whereas it was higher in females when the International Working Group on Sarcopenia (IWGS) criteria were referenced (17% vs. 12%) [22]. Although the prevalence of sarcopenia based on bioelectrical impedance analysis (BIA) was found to be higher than that based on dual-energy X-ray absorptiometry (DXA) in some studies, the BIA method is known to overestimate muscle mass and underestimate fat mass [23,24]. In our study, the BIA method was used and could be the reason for the relatively low prevalence we found. The lack of a single approach in the assessment of sarcopenia causes the prevalence to vary significantly according to the classification and cut-off point used and makes the diagnostic process difficult. The availability of an easily accessible biomarker in the clinic may allow for a single and more objective approach to the diagnosis of sarcopenia. One of the main results of our study was that low serum beta-2 microglobulin levels may be a potential biomarker for the diagnosis of sarcopenia. The muscle regeneration process is driven by intracellular or extracellular matrix components, such as various serum factors and molecules released from damaged muscle tissue, and molecules secreted by immune cells. In sarcopenic muscles, a reduction in the number of myofibres and hypotrophic myofibres occurs, followed by fat and fibrotic tissue infiltrations in the muscles in later stages. There is also a decrease in the number of satellite cells, which are the adult stem cells necessary for the maintenance of skeletal muscle mass [25,26]. These changes result in reduced myoblast dynamics and their ability to repair damaged myofibres [27].

The exact cellular mechanisms behind increased catabolism in myocytes often remain unknown. While myocytes make up the majority of cells in skeletal muscle, they are accompanied by various other cell types with different roles [28]. Age-related obesity and muscle loss (sarcopenia) are closely linked and are mutually influenced by dysfunction in both adipose tissue and skeletal muscle. With ageing, inflammation in adipose tissue prompts fat redistribution to the abdominal area (visceral fat) and into skeletal muscle, leading to reduced strength and functionality. Lipid deposits build up within and between muscle cells, causing mitochondrial dysfunction, disrupting fatty acid β-oxidation, and increasing reactive oxygen species (ROS) production, which results in lipotoxicity, insulin resistance, and the heightened secretion of certain pro-inflammatory cytokines. These cytokines from muscle may further promote adipose tissue degradation and support chronic low-level inflammation [29]. Moreover, oxidative stress, inflammation, mitochondrial dysfunction, reduced protein synthesis, and elevated proteolysis are all essential contributors to muscle atrophy. Notably, oxidative stress plays a central role in initiating skeletal muscle atrophy. It becomes active in the early phases of atrophy and can be influenced by various factors. However, the precise mechanisms by which oxidative stress contributes to muscle atrophy remain to be completely understood [30]. Many different mechanisms are associated with muscle atrophy, and it is understood that muscle regeneration capacity is lower in sarcopenic patients. A biomarker indicating muscle turnover and muscle atrophy would assist in the diagnosis of the disease.

Beta-2 microglobulin is defined as the light protein chain of MHC molecules shed into the serum. The MHC class I antigen is present in most nucleated cells in the human body [28]. Beta-2 microglobulin concentration reflects the rate of cell membrane renewal and cellular turnover, i.e., it is a cell turn-over marker [31]. Decreased muscle mass and decreased regeneration ability of muscle cells may be associated with lower beta-2-microglobulin levels and reduced serum beta-2-microglobulin concentration. In our study, low serum beta-2 microglobulin level was determined as an important predictor of low muscle mass in sarcopenic patients, and beta-2 microglobulin may be a biomarker predicting low muscle regeneration. Kim et al. (2015) showed that each unit increase in beta-2 microglobulin was associated with a decreased risk of severe sarcopenia, but the association was not statistically significant [32]. Beta-2 microglobulin level in body fluids has been used as a biomarker of many diseases. Beta-2 microglobulin is closely linked to various pathological conditions associated with ageing, including malignant diseases. Albumin and haemoglobin were found to be negatively associated with sarcopenia in a meta-analysis of biomarkers for sarcopenia in older persons by Picca et al. [33]. Interleukin-6 was found to be associated with sarcopenia only in people under 75 years of age. Among community-dwelling older adults, those with sarcopenia were found to have higher levels of tumour necrosis factor-alpha than their non-sarcopenic peers. Several metabolic, haematological, and inflammatory biomarkers were associated with sarcopenia. These findings fill an important knowledge gap in the identification of biomarkers for sarcopenia and provide a rationale for biomarker selection in future studies on sarcopenia [33]. The results of our study were important in terms of filling this gap, showing serum beta-2 microglobulin value as a valid test. Sivanathan et al. predicted that a portable and highly efficient beta-2 microglobulin biosensor device will soon be incorporated into screening tests to provide safe, rapid, and reliable test results [34]. This development will also enable the validity and reproducibility of our findings to be quickly checked by researchers.

### Limitations

The use of the bioelectrical impedance method as a muscle mass measurement method, the selection of a hospital-weighted sample, and questions based on patient declaration can be listed as our limitations. The strength of our study is that it is one of few studies that recommend a biomarker for the diagnosis of sarcopenia.

## 5. Conclusions

The results of our study indicate that serum beta-2 microglobulin level is a potential biomarker for the diagnosis of sarcopenia in older adults. Low beta-2 microglobulin levels may be interpreted in favour of sarcopenia. To use serum beta-2 microglobulin level as a potential biomarker in the diagnosis of sarcopenia, our results should be supported by other, more representative, sampling studies, and experimental studies are needed to elucidate the mechanism.

## Figures and Tables

**Table 1:** Distribution of sociodemographic and physical characteristics of the participants according to study group.

Characteristics	Healthy Group (*n* = 238) Mean ± S.D. or *n* (%)	Sarcopenic Group (*n* = 13) Mean ± S.D. or *n* (%)	*p*
Age (years)	71.93 ± 5.87	76.92 ± 8.35	<0.001
Sex: Female/Male	146 (61.3)/92 (38.7)	10 (76.9)/3 (23.1)	0.205
Chronic disease: Present/Absent	195 (81.9)/43 (18.1)	11 (84.6)/2 (15.4)	0.578
Physical activity: Sedentary	238 (100)	13 (100)	N/A
ASM (kg)	21.29 ± 4.26	15.25 ± 2.98	<0.001
ASMI (kg/m^2^)	8.17 ± 6.39	1.12 ± 0.72	<0.001
SARC-F score	5.28 ± 3.64	5.76 ± 3.08	0.640
Hand grip (kg)	23.31 ± 9.02	15.53 ± 5.85	<0.001
Fragility score	2.37 ± 1.36	2.33 ± 1.15	0.916
FFM (kg)	51.86 ± 9.70	39.14 ± 7.35	<0.001
Metabolic age (years)	73.25 ± 9.16	75.69 ± 10.8	0.357
Metabolic rate (kcal)	1.40 ± 0.18	1.22 ± 0.21	<0.001
Internal lubrication	12.97 ± 3.62	9.92 ± 2.81	0.003
Energy (kcal)	1260.30 ± 328.06	1178.16 ± 270.75	0.377
Protein (g)	46.08 ± 15.64	41.90 ± 13.64	0.347
Fat (g)	46.38 ± 14.61	44.63 ± 11.05	0.673
Carbohydrate (g)	159.59 ± 52.80	147.24 ± 46.06	0.410

ASM, Appendicular skeletal muscle; ASMI, Appendicular skeletal muscle index; SARC-F, sarcopenia screening test; FFM, free fat mass (lean body mass); S.D., standard deviation; N/A, not applicable.

**Table 2:** Comparison of serum beta-2 microglobulin levels in case–control group.

**Beta-2** **Microglobulin (mcg/mL)**	**Matched Groups (*n* = 39)**				***p***
	**Normal (*n* = 26)**		**Sarcopenic (*n* = 13)**		
	**Mean ± S.D.**	**Min–Max**	**Mean ± S.D.**	**Min–Max**	
	4.46 ± 3.90	1.38–20.10	1.78 ± 0.46	1.19–2.83	**0.002**

**Table 3:** Serum beta-2 microglobulin level metrics for different cut-offs.

Optimal Cut-Off (mcg/mL)	Sensitivity (%)	Specificity (%)	PPV (%)	NPV (%)	Youden Index	AUC
2.10	84.62	84.62	73.33	91.67	0.692	0.901
2.13	84.62	80.77	68.75	91.30	0.654	0.901
**2.26**	**92.31**	**80.77**	**70.59**	**95.45**	**0.731**	**0.901**
2.53	92.31	76.92	66.67	95.24	0.692	0.901
2.62	92.31	73.08	63.16	95.00	0.654	0.901

PPV, positive predictive value; NPV, negative predictive value; AUC, area under the curve.

**Table 4:** Logistic regression analysis of sarcopenia risk.

	B	*p*	Odds Ratio	95% C.I. for Odds Ratio	
				Lower	Upper
Age (years)	0.122	**0.017**	1.13	1.022	1.249
Sex	−0.095	0.917	0.90	0.151	5.469
Fragility score	0.106	0.731	1.11	0.608	2.032
Energy (kcal/day)	0.009	0.901	1.00	0.872	1.168
Protein (g)	−0.016	0.958	0.98	0.539	1.798
Fat (g)	−0.077	0.907	0.92	0.252	3.402
Carbohydrate (g)	−0.046	0.882	0.95	0.523	1.745
Chronic illness presence	0.587	0.569	1.79	0.239	13.542
Beta-2 microglobulin (risk < 2.26 mcg/mL)	2.375	**0.039**	10.75	1.133	101.993
Constant	−12.550	0.009	0.000		

Note: The analysis was performed for the whole group. Abb. CI: confidence interval, B: beta, *p*: probability.

## Data Availability

The data that support the findings of this study are available on request from the corresponding author. The data are not publicly available due to privacy or ethical restrictions.
